# Supplementary material for: Empirical methods for controlling false positives and estimating confidence in ChIP-Seq peaks
Source: BMC Bioinformatics. 2008 Dec 5;9:523. doi: 10.1186/1471-2105-9-523 (PMC2628906; doi:10.1186/1471-2105-9-523)
Supplement: Additional File 1 — A variety of html documents from the USeq web site detailing the available applications, their best usage, output file type descriptions, command line menus, etc. [file 1471-2105-9-523-S1.zip › USeqUserGuides/JavaDoc.zip!/JavaDoc/util/bio/wrappers/class-use/Primer3Pick.html]

Uses of Class util.bio.wrappers.Primer3Pick


|  |  |  |
| --- | --- | --- |
| **Overview** \| **Package** \| **Class** \| **Use** \| **Tree** \| **Deprecated** \| **Index** \| **Help** | |  |
| PREV   NEXT | **FRAMES**    **NO FRAMES**     **All Classes** |  |


---


## **Uses of Class util.bio.wrappers.Primer3Pick**

| Packages that use Primer3Pick | |
| --- | --- |
| **util.bio.wrappers** | Wrappers for other bio applications. |

Uses of Primer3Pick in util.bio.wrappers

| Methods in util.bio.wrappers that return Primer3Pick | |
| --- | --- |
| `static Primer3Pick[]` | `Primer3Pick.parseFile(java.io.File file)`             Parses a tab delimited file where the sequence to pick primers from is in column 0. |

| Methods in util.bio.wrappers with parameters of type Primer3Pick | |
| --- | --- |
| `java.lang.String` | `Primer3Wrapper.buildExecuteDoc(Primer3Pick[] pp)` |

---


|  |  |  |
| --- | --- | --- |
| **Overview** \| **Package** \| **Class** \| **Use** \| **Tree** \| **Deprecated** \| **Index** \| **Help** | |  |
| PREV   NEXT | **FRAMES**    **NO FRAMES**     **All Classes** |  |


---
